# Prevalence and Characteristics of Hypoxic Hepatitis in COVID-19 Patients in the Intensive Care Unit: A First Retrospective Study

**DOI:** 10.3389/fmed.2020.607206

**Published:** 2021-02-11

**Authors:** Haijun Huang, Hong Li, Shanshan Chen, Xianlong Zhou, Xuan Dai, Jia Wu, Jun Zhang, Lina Shao, Rong Yan, Mingshan Wang, Jiafeng Wang, Yuexing Tu, Minghua Ge

**Affiliations:** ^1^Department of Infectious Disease, Zhejiang Provincial People's Hospital, People's Hospital Affiliated of Hangzhou Medical College, Hangzhou, China; ^2^Medical College of Qingdao University, Qingdao, China; ^3^Graduate School of Clinical Medicine, Bengbu Medical College, Bengbu, China; ^4^Emergency Center, Zhongnan Hospital of Wuhan University, Wuhan, China; ^5^Hubei Clinical Research Center for Emergency and Resuscitation, Zhongnan Hospital of Wuhan University, Wuhan, China; ^6^Hangzhou Medical College, Hangzhou, China; ^7^Department of Hepatobiliary and Pancreatic Surgery and Minimally Invasive Surgery, Zhejiang Provincial People's Hospital, People's Hospital affiliated of Hangzhou Medical College, Hangzhou, China; ^8^Department of Orthopaedic Surgery, Zhejiang Provincial People's Hospital, People's Hospital affiliated of Hangzhou Medical College, Hangzhou, China; ^9^Department of Nephrology, Zhejiang Provincial People's Hospital, People's Hospital affiliated of Hangzhou Medical College, Hangzhou, China; ^10^Department of Head, Neck and Thyroid Surgery, Zhejiang Provincial People's Hospital, People's Hospital affiliated of Hangzhou Medical College, Hangzhou, China; ^11^Department of Intensive Care Unit, Zhejiang Provincial People's Hospital, People's Hospital affiliated of Hangzhou Medical College, Hangzhou, China

**Keywords:** 2019-nCoV, SARS-CoV-2, hypoxic hepatitis, ischemia, liver injury, multiple organ failure (MOF), COVID-19

## Abstract

**Purpose:** Coronavirus disease 2019 (COVID-19) has been associated with acute liver injury in reports worldwide. But no studies to date have described hypoxic hepatitis (HH) in patients with COVID-19. We aim to identify the prevalence of and possible mechanisms of HH in COVID-19 patients in the Intensive Care Unit (ICU).

**Methods:** This retrospective study was conducted on 51 patients with confirmed SARS-CoV-2 infection in the ICU at Zhongnan Hospital of Wuhan University from December 21, 2019, to March 11, 2020. Information on clinical features of enrolled patients was collected for analysis.

**Results:** HH was observed in 5.88% of the ICU patients with SARS-CoV-2 infection. All HH patients were progressing to respiratory failure and peak alanine aminotransferase (ALT) values were 1665, 1414, and 1140 U/L during hospitalization, respectively. All patients with HH died as a result of the deterioration of multiple organ failure (MOF). The dynamic changes of ALT, aspartate transaminase (AST), and total bilirubin (TBIL) levels were more dramatic in HH groups. Levels of TBIL, C-reactive protein (CRP), procalcitonin (PCT), and interleukin-6(IL-6) showed statistically significant elevation in HH cases compared with that in non-HH cases (*P* < 0.001). Besides, the median survival time of the HH group was significantly shorter than the non-HH group (*P* < 0.05).

**Conclusions:** In ICU, HH was not a rare condition in patients with severe COVID-19 and has a high mortality. The main causes of HH are respiratory and cardiac failure and may be associated with the immune-mediated inflammatory response. Clinicians should search for any underlying hemodynamic or respiratory instability even in patients with normal ALT levels on admission.

## Introduction

In December 2019, a range of viral pneumonia cases started to spread in the city of Wuhan, China. On 11 February 2020, this unknown etiology was officially named severe acute respiratory syndrome coronavirus 2(SARS-CoV-2) (1), and the disease was named coronavirus disease 2019(COVID-19) by the World Health Organization. Some patients had different degrees of liver injury. Moreover, four relatively large-scale studies have reported the clinical characteristics of COVID-19 patients, including liver impairment (2–5). Although transaminase elevation is usually mild, severe liver injury has been reported (3–6). However, the reason for severe liver injury is still not clear.

Acute liver impairment can present as a life-threatening disorder and can be caused by a series of reasons, including drug-induced liver injury (DILI), acute viral hepatitis, HH, acute alcohol-induced liver injury, liver trauma, etc. Whether SARS-CoV-2 is hepatotropic like viral hepatitis A, B, C, and what roles does the virus plays in the course of liver injury is worth studying. What's more, other causes of acute liver injury, if not SARS-CoV-2, need to be identified.

HH is the most common cause of a significant but transient elevation in serum aminotransferase activities (S-AT) in most studies. In ICU, the prevalence of HH accounts for at least 1% of admission (7). Currently, the pathogenesis of HH is not very clear, but most scholars agree with the three mechanisms proposed by Dunn et al. (8) that lead to HH: hepatic ischemia caused by reduced blood flow to the liver, venous congestion caused by right heart failure, and arterial hypoxemia caused by decreased blood oxygen content. Thus, remarkedly hypoxemic conditions could result in HH. Severe COVID-19 cases have led to respiratory failure, which can decrease oxygen supply to the liver. For this reason, HH may be a possible cause of severe liver injury in COVID-19 patients. Up to date, there has been no study of HH in patients infected with SARS-CoV-2. In the largest cohort study of critically ill patients with HH, the 28-day mortality in ICU was 45.0%(9). Because of this high mortality, the prompt identification and treatment of HH are crucial to the prognosis. We aim to describe the prevalence of HH in COVID-19 patients in ICU, as well as possible mechanisms.

## Materials and Methods

### Patients

From December 21, 2019, to March 11, 2020, 51 patients with COVID-19 were admitted and treated to the ICU at Zhongnan Hospital of Wuhan University. Epidemiological, clinical, laboratory characteristics, treatment, and outcomes data were acquired from the electronic medical records. This retrospective study was approved by the Research Ethics Commission of Zhongnan Hospital of Wuhan University and the Ethics Committee of Zhejiang Provincial People's Hospital. Due to the retrospective nature of this study, the need for informed consent was waived.

### Data Collection

Laboratory investigations included complete blood count, coagulation function, routine biochemical, and liver function tests. Parameters related to patient characteristics included sex, age, and comorbidities (diabetes mellitus, hypertension, coronary heart disease, heart disease, and chronic obstructive pulmonary disease). Parameters related to the episode of HH included cause, supportive therapy (vasopressor agents, mechanical ventilation, and extracorporeal membrane oxygenation), and specific drug use (ribavirin, remdesivir, oseltamivir, glucocorticoid, etc.).

### Hypoxic Hepatitis

Patients who met all of the following criteria were diagnosed with HH based on a previous report (10) (i) a massive but transient elevated ALT level [more than 20-fold the upper limit of normal (ULN)], (ii) the presence of respiratory, cardiac or circulatory failure, and (iii) exclusion of other causes of liver injury. Liver biopsy was not required for the diagnosis of HH, in agreement with other studies showing that a histological confirmation is unwarranted and even inadvisable when the criteria listed above are met (11, 12).

### Statistical Analysis

Statistical analysis was performed using the SPSS software package (version 21.0, SPSS Inc., IBM, Chicago, IL, USA). Categorical variables were described as frequency and percentages, and continuous variables as means ± standard deviation (SD) or median and interquartile range (IQR). Continuous variables were compared using independent group *t*-tests when the data were normally distributed; otherwise, the Mann-Whitney U test was used. Comparison of categorical variables was done using the chi-square test or the Fisher exact test if the cell counts were small. Dynamic changes of liver function indicators by the influence of hypoxic hepatitis were presented using locally weighted scatterplot smoothing (LOESS). Survival analysis was performed by the Kaplan-Meier and Log rank test. A *P*-value < 0.05(two-tailed) was considered to indicate significance.

## Results

### Characteristics of the Patients With COVID-19 in ICU

The 51 patients in ICU with COVID-19 were predominantly male (*n* = 35,68.63%), and aged ≤65 years (*n* = 32,62.75%). There underlying medical conditions described in Table 1, mainly included hypertension (*n* = 22,43.14%), diabetes mellitus (DM) (*n* = 7,13.73%), coronary heart disease (CHD) (*n* = 12,23.53%), chronic obstructive pulmonary disease (COPD) (*n* = 2,3.92%). None of the critical patients had a malignant tumor. The proportion of patients with liver impairment on admission was much lower than the hospitalization (*n* = 39, 76.47% vs. *n* = 21, 41.18%, *P* < 0.001). Antiviral therapy was performed in 38 (74.51%) patients and antibiotic therapy was performed in 16 (31.4%), while the combination of the two therapies was 11 (21.56%). What's more, the proportion of patients using two or more antiviral drugs is 23.53% (12/51) and glucocorticoid therapy is 82.35% (42/51). Mechanical ventilation was performed in 37 (72.55%) while extracorporeal membrane oxygenation (ECMO) was performed in 3 (5.88%) (Table 1).

**Table 1:** Characteristics of the patients with COVID-19 in ICU (*n* = 51).

**Characteristics**	**Number (%)**	
**Age**		
≤65 years	32 (62.75)	
>65 years	19 (37.25)	
**Sex**		
Male	35 (68.63)	
Female	16 (31.37)	
BMI>25 (kg/m^2^)	16 (31.37)	
**Underlying medical conditions**		
Hypertension	22 (43.14)	
DM	7 (13.73)	
CHD	12 (23.53)	
COPD	2 (3.92)	
Cerebrovascular disease	5 (9.80)	
Hepatopathy	1 (1.96)	
Hemorrhage of digestive tract	2 (3.92)	
Chronic kidney disease	1 (1.96)	
Renal transplant	1 (1.96)	
Malignant tumor	0	
**Liver impairment**		Value (mean ± SD) U/L
On admission	21 (41.18)	53.13 ± 65.55
ULN <ALT ≤20-fold ULN	21 (41.18)	53.13 ± 65.55
ALT >20-fold ULN	0	
Hospitalization^*^	39 (76.47)^*^	70.54 ± 142.90
ULN <ALT ≤20-fold ULN	36 (70.59)	118.23 ± 90.75
ALT >20-fold ULN	3 (5.88)	1406.33 ± 262.58^#^
**Treatment**		
Antibiotic therapy	16 (31.37)	
Antiviral therapy	38 (74.51)	
A combination of antibiotic and antiviral therapy	11 (21.56)	
Multiple antiviral therapies (≥2)	12 (23.53)	
Glucocorticoid therapy	42 (82.35)	
Mechanical ventilation	37 (72.55)	
ECMO	3 (5.88)	

* *Liver impairment ratio (hospitalization vs. on admission, P < 0.001)*.

# *Peak ALT level during hospitalization (HH patients vs. non-HH patients with liver impairment, P < 0.001)*.

*ALT, alanine aminotransferase; BMI, body mass index; CHD, coronary heart disease; COPD, chronic obstructive pulmonary disease; DM, diabetes mellitus; ULN, upper limit of normal; ECMO: extracorporeal membrane oxygenation; SD, standard deviation; HH, hypoxic hepatitis*.

An ALT level of >20-fold the ULN was found in only three patients, not on admission but during hospitalization. The peak value of ALT was 1665, 1414, and 1140 U/L respectively. The degree of ALT elevation in HH patients was significantly higher than that in non-HH patients with liver impairment (during hospitalization, 1406.33 ± 262.58 vs. 118.23 ± 90.75 U/L, *P* < 0.001). These cases were diagnosed as HH. The incidence of HH in our single-center cohort of ICU patients with COVID-19 was 5.88% (3/51). All cases deteriorated into respiratory failure. Also, viral hepatitis and autoimmune diseases were excluded based on negative results of serum marker tests.

### Comparison of Clinical Characteristics Between HH and Non-HH Patients

**Figure 1:**
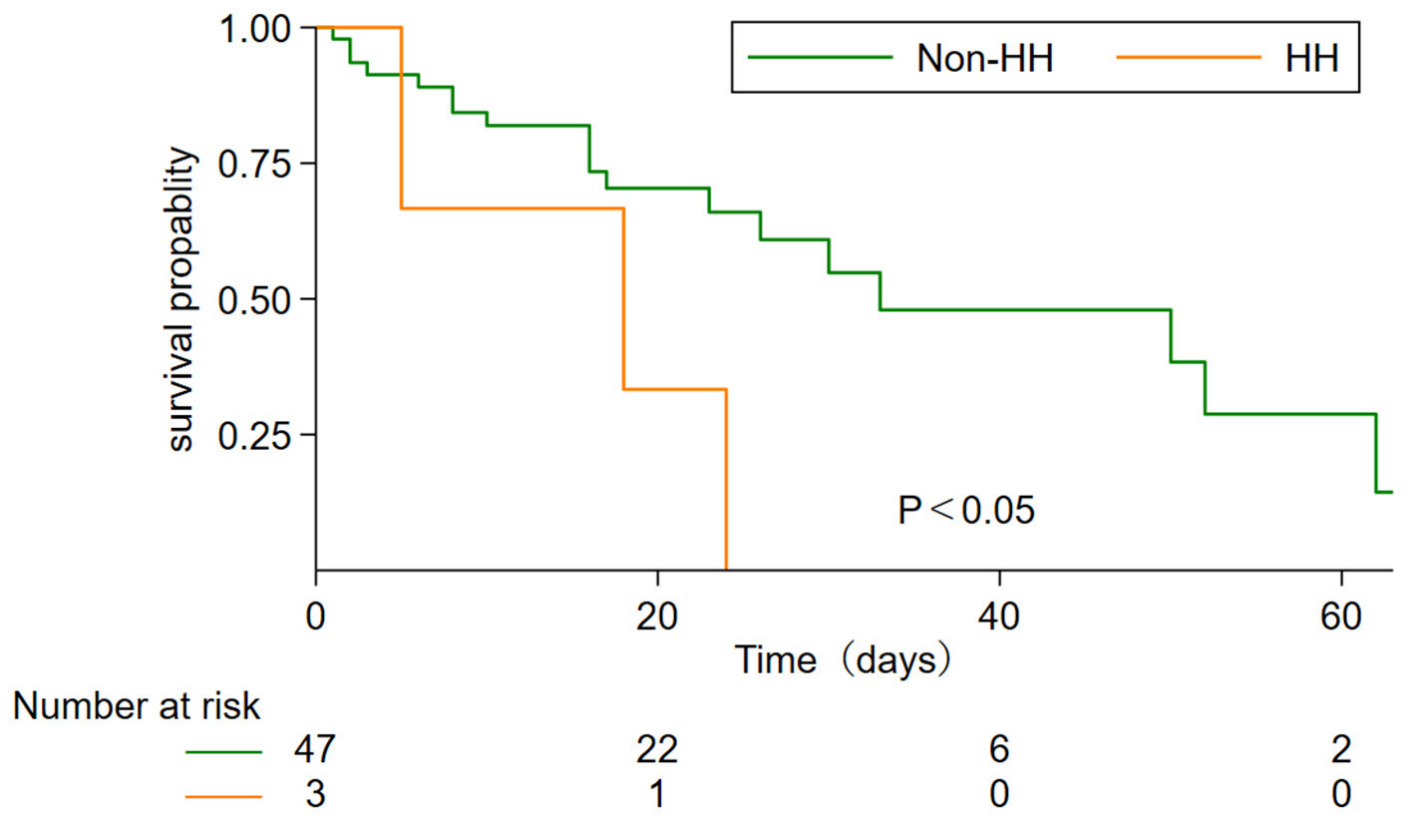
Comparison of survival probability between HH and non-HH group. Group comparisons were performed using the Kaplan-Meier and long-rank test.

The clinical characteristics of the two groups were shown in Table 2. Mean age at diagnosis was (63.33 ± 7.64), which was slightly older than that non-HH group. There was no significant difference in age and sex between the two groups. In our cohort of 51 patients, no children or adolescents were infected. The incidence of hypertension and CHD were 2 (66.67%) and 1 (33.33%), respectively, which were higher than that of 20 (41.67%) and 11 (22.92%) patients without HH. There were no DM and hepatopathy in the HH group, compared with 7/48 (14.58%) cases of DM and 1/48 (2.08%) cases of hepatopathy in the non-HH group. The most common symptoms at the onset of illness were fever (88.23%), cough (64.71%), and dyspnea (52.94%). There was no significant difference in the incidence of myalgia and COPD between the two groups. To get the outcomes of the patients, we followed up to April 5, 2020, 29 (60.42%) patients have been discharged and 19 (39.58%) died in the group of non-HH patients, while 3 (100.00%) patients have all died in the group of HH. Besides, Figure 1 also showed that the median survival time of the HH group was significantly shorter than the non-HH group (*p* < 0.05). Fitness for discharge was based on abatement of fever for at least 10 days, with the improvement of chest radiographic evidence and viral clearance in respiratory samples from the upper respiratory tract (Table 2).

**Table 2:** Clinical characteristics of COVID-19 patients.

**Characteristics**	**With HH (*n* = 3)**	**Without HH (*n* = 48)**	***P* value**
Age (years)	63.33 ± 7.638	59.94 ± 14.110	0.68
Sex (male)	3 (100.00)	32 (66.67)	0.54
Current smoker (%)	2 (66.67)	9 (18.75)	0.22
Current drinker (%)	1 (33.33)	10 (20.83)	>0.999
**Pre-existing conditions (%)**			
Hypertension	2 (66.67)	20 (41.67)	0.81
DM	0	7 (14.58)	>0.999
Heart disease	1 (33.33)	11 (22.92)	>0.999
COPD	1 (33.33)	1 (2.08)	0.24
Hepatopathy	0	1 (2.08)	>0.999
Other diseases	3 (100.00)	20 (41.67)	0.09
Comorbidities	3 (100.00)	32 (66.67)	0.543
**Symptom (%)**			
Fever	3 (100.00)	42 (87.50)	>0.999
Cough	3 (100.00)	30 (62.50)	0.54
Dyspnea	3 (100.00)	24 (50.00)	0.24
Myalgia	3 (33.33)	8 (16.67)	0.25
Chest distress	3 (100.00)	22 (45.83)	0.11
Other	2 (66.67)	17 (35.42)	0.64
**Outcome (%)**			
Discharge	0	29 (60.42)	0.07
Death	3 (100.00)	19 (39.58)	0.07

### Selected Laboratory Tests of COVID-19 Patients

Table 3 shows that the median values of lymphocytes and PLT were 0.26 (0.20–0.48) and 64.00 (48.25–147.25), respectively, which were significantly lower than those of patients without HH (*P* < 0.001). No significant difference in the level of white blood cells or hemoglobin between the two groups was observed (*P* > 0.05). In indexes of liver enzymes and function, patients with HH had significant degrees of ALB reduction compared with that non-HH group (*P* < 0.05) and the concentration of ALB has reached the level of hypoalbuminemia (ALB<30 g/L). The median levels of ALT, AST, γ-GT, LDH, and AKP in patients with HH were no significant differences between the two groups. Furthermore, ALT, AST, and γ-GT were slightly above normal, while LDH was significantly above normal. No obvious jaundice was observed in any of the patients with HH, even though the median TBIL level in the HH group was significantly higher than that non-HH group (*P* < 0.001). Since the coagulation proteins required for blood coagulation cascade are mainly produced by the liver, and the half-life of coagulation factors is shorter than ALB, INR is often used as the marker of liver synthesis function (13). Patients with HH had higher median INR values compared with those patients without HH (1.26 vs. 1.22), though it presented only a very slight INR elevation. Inflammatory markers among these critically ill patients, including CRP, PCT, D-dimer, and IL-6. The concentration of PCT, CRP, and IL-6 were 5.43 (1.87–20.13), 235.30 (102.80–300.50), and 356.10 (149.70–1622.25), respectively, which were significantly higher than those of patients without HH (*P* < 0.001). Besides, CRP as the indicator of acute inflammation, the levels of CRP in both groups was well above normal. As for the indicator of renal function, the median value of creatinine was 88.50 (71.00–124.55) and 70.00 (52.53–115.70), respectively, which were significantly higher than that non-HH group (*P* < 0.05), although still in the normal range. The levels of myocardial necrosis markers in patients with HH were also higher than those in patients without HH and far above normal.

**Table 3:** Selected laboratory data of COVID-19 patients.

**Characteristics**	**With HH (*n* = 3)**	**Without HH (*n* = 48)**	***P*-value**
**BLOOD COUNTS**			
WBC (× 10^9^/L)	9.98 (6.19–15.23)	9.32 (6.58–14.10)	0.89
Lymphocyte (× 10^9^/L)	0.26 (0.20–0.48)	0.57 (0.34–1.04)	<0.001
Hgb (g/L)	95.95 (83.90–123.70)	111.00 (82.85–126.90)	0.26
PLT (× 10^9^/L)	64.00 (48.25–147.25)	151.00 (103.00–219.50)	<0.001
**LIVER ENZYMES AND FUNCTION**			
ALB (g/L)	28.95 (24.95–31.28)	30.70 (27.80–34.35)	0.02
ALT (U/L)	42.00 (21.00–54.00)	40.00 (26.00–69.00)	0.50
AST (U/L)	40.00 (34.00–61.00)	38.00 (26.00–65.00)	0.23
TBIL (μmol/L)	21.30 (14.50–32.90)	14.20 (10.20–21.10)	<0.001
γ-GT (U/L)	70.50 (43.75–97.00)	45.00 (24.00–111.00)	0.07
ALP (U/L)	100.00 (87.25–120.75)	90.50 (71.00–122.75)	0.13
LDH (U/L)	432.00 (347.25–895.25)	460.50 (322.25–641.75)	0.86
INR	1.26 (1.16–1.43)	1.22 (1.13–1.36)	0.14
**ADDITIONAL MARKERS OF ORGAN DAMAGE AND INFLAMMATION**			
CRP (mg/L)	235.30 (102.80–300.50)	53.60 (23.58–112.33)	<0.001
PCT (ng/ml)	5.43(1.87–20.13)	0.29(0.06–1.90)	<0.001
D-dimer (μg/L)	935.00 (622.50–2049.00)	1123.00 (578.00–3254.00)	0.61
IL-6 (pg/mL)	356.10 (149.70–1622.25)	45.21 (17.90–103.60)	<0.001
Creatinine (μmol/L)	88.50 (71.00–124.55)	70.00 (52.53–115.70)	0.02

*WBC, white blood cell;Hgb, hemoglobin; PLT, platelet count; ALB, albumin; ALT, alanine aminotransferase; AST, aspartate transaminase; TBIL, total bilirubin; γ-GT, γ-glutamyl transpeptidase; ALP, alkaline phosphatase; LDH, lactic dehydrogenase; INR, international normalized ratio; CRP, C-reactive protein; PCT, procalcitonin; IL-6, interleukin-6*.

### Dynamic Changes in Liver Function Indicators in COVID Patients in ICU

**Figure 2:**
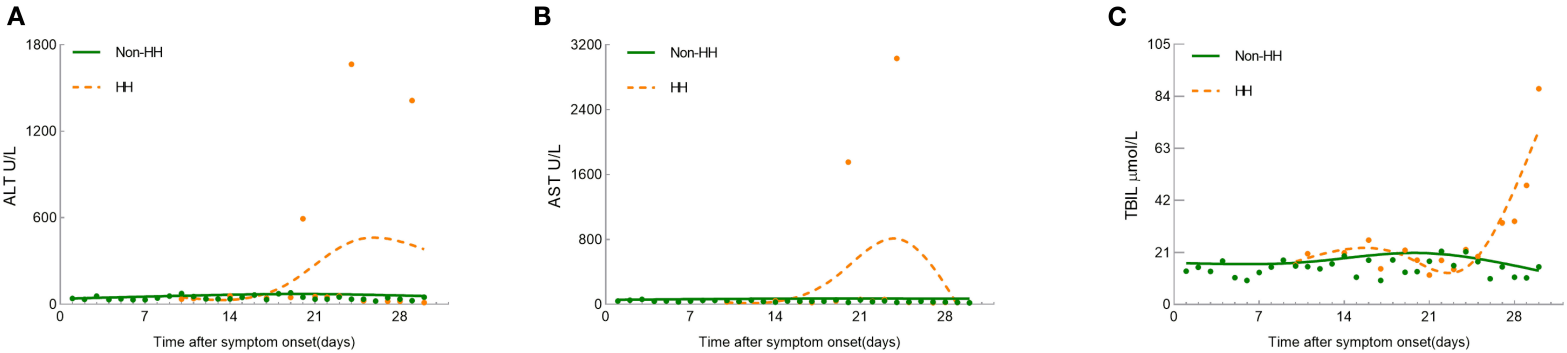
Smooth trajectories and scatters represent the median values **(A)** ALT; **(B)** AST; **(C)** TBIL in the HH and non-HH groups. The ULN in our health care system is defined as 40 U/L for ALT, 40 U/L for AST, and 21 μmol/L for TBIL, respectively.

To determine the trajectory of liver function indicators in patients enrolled in this study, multiple liver function test results were recorded during hospitalization. LOESS models illustrated the trajectory of ALT, AST, and TBIL between the two groups (Figure 2). From the general trend, the rangeability of the non-HH group was much flatter than that of the HH group. Unlike the non-HH group, the curve of HH started on day 10. Figure 2A suggested that the curve of the non-HH group fluctuated roughly in a range of 1–2 times of ULN. ALT began to rise rapidly at the 2nd week and peaked within 3 to 4 weeks after symptom onset in the HH group. Besides, invasive mechanical ventilation was used when ALT levels reached a peak. Figure 2B illustrated that the curve of the non-HH group is close to a straight line compared with the HH group. Similar to ALT, AST also increased dramatically at the 2nd week and peaked within 3 to 4 weeks. Subsequently, the curve dropped sharply to a normal level. Figure 2C showed that the fluctuation in TBIL levels was mild and normal in the non-HH group. Moreover, TBIL levels were slightly higher than that non-HH group in the 1st week of the curve. Shortly after TBIL slowed down in the next week. However, the curve of TBIL was increased dramatically in the 3rd week. The dynamic changes in indicators suggested potential mechanisms of COVID-19 patients with HH in ICU.

## Discussion

HH, as manifested by ALT abrupt and massive elevation, is emerging as a clinical consequence of COVID-19, and often predict a poor outcome. These severely infected patients with HH had a high rate of ARDS and a high risk of death. SARS-CoV-2 infection in humans can cause respiratory diseases, acute kidney injury, myocarditis, thrombosis, and acute liver injury (14). Most studies reported the prevalence of liver enzyme elevation has ranged from 20 to 30% and severe liver injury was uncommon (5, 15). However, extreme liver impairment (ALT >20-fold the ULN) was not rare in our study (*n* = 3, 5.88%) compared with previous reports (7). What's more, the significant difference of ALT elevation between HH patients and non-HH patients with liver impairment (*P* < 0.001) indicated that HH can be distinguished from other types of liver injury by the sharp increase of ALT level. It is also reported that liver impairment is caused by drug hepatotoxicity (such as remdesivir) (16) and immune-mediated inflammation (17). Thus, it's necessary to distinguish the causes of liver impairment.

DILI can be ruled out because hepatotoxic drugs continue to be used, and ALT also gradually decreases. Autoimmune hepatitis (AIH) was excluded because the score is not up to the diagnostic criteria(include autoantibodies, immunoglobulins, viral markers, and histological findings) proposed by the International Autoimmune Hepatitis Group (18). No imaging evidence suggested hepatic steatosis and no patients met one of the following clinical parameters, such as being overweight, having type 2 diabetes mellitus, or exhibiting metabolic dysregulation (19). Thus, metabolic associated fatty liver disease (MAFLD) was also ruled out. Henrion et al. reported that heart failure, respiratory failure, and septic toxic shock accounted for more than 90% of HH cases and the core mechanism was reduced oxygen supply to the liver (7). The hallmark of COVID-19 is respiratory failure. HH is therefore frequent in severe cases. In our study, 3 HH cases all progressed to respiratory failure, which led to hypoxia of liver tissue and abnormalities of liver function. Moreover, heart failure reduces the output, thereby decreasing the blood flow to the liver and further exacerbating hypoxia in the liver. This is consistent with previous research (7, 8). Although several hemodynamic mechanisms of liver hypoxia are involved, liver ischemia is not the only explanation for HH. On the one hand, previous studies indicated that ALT levels correlate well with markers of inflammation and are likely higher among patients with severe cytokine release syndromes (14, 20, 21). On the other hand, cytokine storm syndromes have been described in COVID-19, and are associated with severe elevations in liver enzymes (22). What's more, IL-6 is significantly increased in severe COVID-19 patients and plays a key role in the so-called “cytokine storm” (23, 24). In our study, the levels of ALT were extremely elevated and the concentrations of inflammatory markers including CRP, D-dimer, and IL-6 were significantly far above normal. Therefore, it suggested that HH may be associated with the immune-mediated inflammatory response.

Ours is the first batch of treatment centers for COVID-19 patients in the world. COVID-19 patients in ICU accounted for 7.02% (51/726) of the total number of patients diagnosed with SARA-CoV-2 infection during our observation. The prevalence of HH in patients with severe SARS-CoV-2 infection in our ICU is 5.88%. Most studies have reported an incidence of 0.9–2.4% (7). This is lower than ours, which indicated that patients with severe SARS-CoV-2 infection in ICU have a higher incidence of HH than that of patients with other causes.

At present, there has been no study on the dynamic changes of liver function in patients with COVID-19 combined with HH, and only a few studies in COVID-19-related liver injury (25, 26). The increase of ALT and AST levels in the early stage of the disease might be related to the immune-mediated inflammation in the liver. However, when ALT and AST showed a downward trend in the late stage of the disease in the HH group, the level of TBIL increased sharply. This may be a result of MOF.

The prognosis of HH is poor. Extensive analysis by Aboelsoud et al. showed that the mortality of HH was 44.1% (27). However, the mortality rate in our study was as high as 100%. MOF contributed heavily to the high mortality. We also observed an elevation in INR, significant hypoalbuminemia, and lymphopenia in patients with HH. Hypoalbuminemia is emerging as a consistent risk factor for severe disease (3, 6, 28) and is linked to poor clinical outcomes for hospitalized patients (29). Besides, increased INR in severe COVID-19 patients indicate damage of liver synthetic function. A study has also shown that lymphopenia may be a key factor related to disease severity and mortality (30). All of these factors can affect the prognosis of HH. In general, severe liver injury was not the direct cause of death. These patients died as a consequence of MOF, but the occurrence of HH had a high impact on the mortality of those critically ill patients.

In conclusion, we report a 5.88% prevalence of HH in COVID-19 patients in ICU, the first study to combine COVID-19 and HH. HH was not a rare condition in ICU, and was frequently accompanied by MOF, with high mortality. Patients with COVID-19 developed MOF accompanied by a sudden and sharp elevation of serum ALT level during hospitalization, which should be considered HH. Attention should also be paid to monitor liver function during the course of COVID-19, especially in patients with higher disease severity.

## Data Availability Statement

All data generated or analyzed during this study are included in this manuscript.

## Ethics Statement

The analysis was approved by the Research Ethics Commission of Zhongnan Hospital of Wuhan University and the Ethics Committee of Zhejiang Provincial People's Hospital, and the need for informed consent was waived.

## Author Contributions

MG and YT designed study and revised the manuscript. HH and XZ analyzed data and prepared the manuscript. HL analyzed data and performed manuscript drafting. SC performed manuscript drafting. XD arranged and filtered data. JW and JZ searched the literature and analyzed data. LS, RY, MW, and JW collect data. HH reviewed the results and made critical comments on the manuscript. All authors contributed to the article and approved the submitted version.

## Conflict of Interest

The authors declare that the research was conducted in the absence of any commercial or financial relationships that could be construed as a potential conflict of interest.

## Abbreviations

HH: hypoxic hepatitis
SARA-CoV-2: severe acute respiratory syndrome coronavirus 2
COVID-19: coronavirus disease 19
ICU: intensive care unit
CHD: coronary heart disease
COPD: chronic obstructive pulmonary disease
DM: diabetes mellitus
ECMO: extracorporeal membrane oxygenation
SD: standard deviation
ULN: upper limit of normal
BMI: body mass index
ALT: alanine aminotransferase
AST: aspartate aminotransferase
TBIL: total bilirubin
γ-GT: γ-glutamyl transpeptidase
ALP: alkaline phosphatase
LDH: lactate dehydrogenase
CRP: C-reactive protein
IL-6: interleukin-6
INR: international normalized ratio
PCT: procalcitonin
Hgb: hemoglobin
WBC: white blood cell
PLT: platelet count
ALB: albumin
AIH: autoimmune hepatitis
MAFLD: metabolic associated fatty liver disease.
